# Effect of cerebral vasomotion during physical exercise on associative memory, a near-infrared spectroscopy study

**DOI:** 10.1117/1.NPh.4.4.041404

**Published:** 2017-07-25

**Authors:** Blanca Marin Bosch, Aurélien Bringard, Guido Ferretti, Sophie Schwartz, Kinga Iglói

**Affiliations:** a University of Geneva, Faculty of Medicine, Department of Neuroscience, Geneva, Switzerland; b Geneva University Hospitals, Department of Anesthesiology, Pharmacology, and Intensive Care, Geneva, Switzerland; c University of Geneva, Swiss Center for Affective Neurosciences, Geneva, Switzerland; d University of Geneva, Geneva Neuroscience Center, Geneva, Switzerland

**Keywords:** near-infrared spectroscopy, vasomotion, associative memory, hippocampus, prefrontal cortex

## Abstract

Regular physical exercise has been shown to benefit neurocognitive functions, especially enhancing neurogenesis in the hippocampus. However, the effects of a single exercise session on cognitive functions are controversial. To address this issue, we measured hemodynamic changes in the brain during physical exercise using near-infrared spectroscopy (NIRS) and investigated related effects on memory consolidation processes. Healthy young participants underwent two experimental visits. During each visit, they performed an associative memory task in which they first encoded a series of pictures, then spent 30-min exercising or resting, and finally were asked to recall the picture associations. We used NIRS to track changes in oxygenated hemoglobin concentration over the prefrontal cortex during exercise and rest. To characterize local tissue oxygenation and perfusion, we focused on low frequency oscillations in NIRS, also called vasomotion. We report a significant increase in associative memory consolidation after exercise, as compared to after rest, along with an overall increase in vasomotion. Additionally, performance improvement after exercise correlated positively with power in the neurogenic component (0.02 to 0.04 Hz) and negatively with power in the endothelial component (0.003 to 0.02 Hz). Overall, these results suggest that changes in vasomotion over the prefrontal cortex during exercise may promote memory consolidation processes.

## 1 Introduction

Over the past decades, public policies have reiterated exercise recommendations, with a special emphasis placed on the benefits of physical activity on general health. Yet, regular physical exercise has also been shown to have a positive impact on cognition,^1^^,^^2^ notably in the memory domain.^3^

Experimental evidence in rodents has accumulated to support the claim that regular exercise improves brain plasticity and cognition by promoting neurogenesis and cell proliferation in the adult hippocampus,^4^^–^^6^ as well as dendritic length and complexity.^7^ In humans, it has been suggested that regular physical exercise enhances hippocampal and entorhinal gray matter density,^8^^,^^9^ and hence improves memory functions.^10^ But the effects of an acute bout of physical exercise on memory remain controversial, with some studies reporting positive effects^11^ and others reporting little or no effects,^12^ especially in meta-analyses.^3^^,^^13^^,^^14^ The brain mechanisms underlying the effects of exercise on memory processes is also a debated issue. For example, Basso et al.^15^ suggested that performance enhancement related to physical exercise is primarily linked to prefrontal and not hippocampal function, whereas a recent study using functional magnetic resonance imaging (fMRI) after acute physical exercise revealed changes in hippocampal memory representations.^11^

Crucially, little is known about what is happening in the brain during physical exercise although this is when plasticity mechanisms should be modulated. Imaging brain activity using fMRI or electroencephalography (EEG) during exercise is problematic because of movement-related signal distortion. However, near-infrared spectroscopy (NIRS) is a method that has proved to be resistant to movement artifacts^16^ and may thus be an ideal tool to measure cerebral hemodynamic changes during physical exercise.

In addition to measuring standard hemodynamic changes, NIRS also allows measuring vasomotion-induced oscillations, which correspond to spontaneous low-frequency (0.003 to 0.15 Hz) rhythmic changes in the diameter of small vessels, see Aalkjaer et al.^17^ for review. Increased vasomotion was shown to lead to improved perfusion^18^^,^^19^ and local tissue oxygenation.^20^^,^^21^ Changes in vasomotor oscillations have a functional significance because they have been linked to cognitive load,^22^ and also to the onset of Alzheimer’s disease.^23^ Vasomotion can be divided into three frequency subbands, according to the origin of the oscillations.^24^ Low-frequency oscillations ranging from 0.04 to 0.15 Hz can be linked to the activity of smooth muscles of arterioles, thus called myogenic component.^25^ Lower frequency oscillations ranging from 0.02 to 0.04 Hz can be attributed to the intrinsic nervous activity and are referred to as neurogenic component.^26^ Ultralow oscillations between 0.003 and 0.02 Hz may reflect endothelial activity and mediated by the release of nitric oxide, henceforth named endothelial component.^25^^,^^27^

In this study, we used NIRS during an acute bout of medium intensity physical exercise or quiet rest to test whether exercise enhances memory consolidation processes and how such effects on memory may implicate changes in oxygenated hemoglobin (${\mathrm{HbO}}_{2}$) and deoxygenated hemoglobin (HHb) over the prefrontal cortex (the function of which may be improved by acute exercise^15^).

## 2 Materials and Methods

### 2.1 Participants

Seventeen healthy volunteers (10 females) between the ages of 21 and 39 (mean age of 29±5.7) participated in this study. All volunteers gave written informed consent and received financial compensation for their participation in this study, which was approved by the Ethics Committee of the Geneva University Hospitals. All included participants were right-handed, nonsmokers, free from psychiatric, and neurological history reported performing aerobic exercise regularly (e.g., running, cycling, or swimming) at least twice a week and had a normal or corrected-to-normal vision. The participants were requested to refrain from all caffeine and alcohol-containing beverages and intense physical activity for the 48 h preceding the experiment. All participants first came for an introductory session to get acquainted with the task, and then underwent two experimental sessions, according to a randomized, crossover design (each visit was separated by at least 3 days). Three participants were excluded from the analyses: one participant was performing at chance level in the memory task and for two participants, NIRS data were not exploitable.

### 2.2 Experimental Procedure

**Fig. 1:**
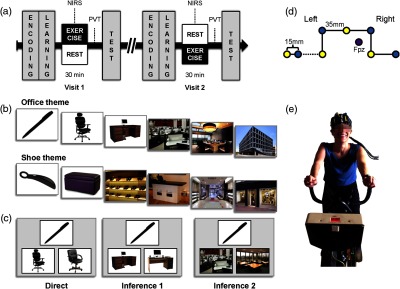
Procedure. (a) Experimental design. All participants underwent two experimental visits composed of an encoding and learning sessions of the associative memory task followed by either 30 min of medium intensity exercise or 30 of quiet rest. Then all participants performed a PVT task and the test part of the associative memory task. (b) Associative memory task: example of one series of the office and the shoe theme. Themes were counterbalanced between first and second visits and the exercise and rest conditions. (c) Test part of the associative memory task, example of direct, inference 1, and inference 2 trials. (d) Schematic representation of the optode template used for NIRS measurements, solid lines show measured channels, the dashed line shows an excluded channel as it is crossing a main facial vessel. Interoptode distance is 35 mm except for short channel at 15 mm. Fpz was localized according to standard 10-20 EEG system. (e) Example of participant during the exercise condition wearing NIRS.

We adapted an associative memory task^28^ consisting of two parts: training (subdivided into encoding and learning) and test, separated by an exercise or rest period [Fig. 1(a)]. For each experimental visit, the participants first performed the encoding and learning parts of the memory task. Then, we placed the NIRS optodes over their prefrontal cortex [Figs. 1(d) and 1(e)] before they exercised (on a cycle ergometer) or rested for 30 min. After 10 min of recovery, during which their vigilance level was assessed [psychomotor vigilance task (PVT)],^29^ the participants performed the test part of the memory task. During the introductory session, all participants performed a shortened version of the associative task (encoding, learning, and test).

To avoid interference and learning across visits, we designed three versions of the memory task, one shortened version for the introductory session, and two full versions for the experimental visits. The experimental visits’ versions were matched in difficulty and counterbalanced across the exercise condition (exercise and rest) and the visits (first and second) [see Fig. 1(b)]. During each experimental visit, the participants had to memorize eight different series containing six pictures belonging to a specific theme: “office” or “shoe shop” (one theme per visit). For the introductory session, the participants had to memorize five series of a “swimming pool” theme.

#### 2.2.1 Encoding

The participants were shown each of the eight series of pictures once, one picture at a time (2000 ms per picture) and were asked to memorize each sequence as a whole.

#### 2.2.2 Learning

For each series, the first picture was shown [pen, for the “office” theme in this example; Fig. 1(b)] followed by the same picture with two options for the second picture in the series (chair), one being the correct next picture and the other picture being from a different series. The participants had to select the correct next picture by pressing a right or left button. The correct picture was then shown (providing a feedback for each trial), followed by this same picture together with the two next options for the third picture in the series (desk). This continued until the last picture in the series (office building). Learning comprised three blocks, each with all eight series shown once.

#### 2.2.3 Exercise and rest conditions

For the exercise condition, the participants pedaled on a cycle ergometer (Ergoline GmbH, Bitz, Germany) at a 50-W power. The pedaling frequency was kept between 60 and 80 cycles per minute, which was shown on a small screen in front of the participant. For the rest condition, the participants were to sit on a chair and were allowed to quietly read magazines. Both exercise and rest conditions lasted for 30 min. The participants were wearing a Polar RS800CX N device to measure heart rate throughout both experimental visits. Since the experimental exercise intensity was identical for all participants, we could derive estimates of individual fitness levels from heart rate.^30^ This was done by first calculating the theoretical maximal heart rate for each participant based on the age adjusted maximal heart rate (AAMHR) formula.^31^ Based on the assumption that a higher fitness level results in a lowered heart rate (for a given exercise intensity—50 W here—and young participants), we computed the percentage of AAMHR to which each participant’s mean heart rate corresponded. This measure provided an estimate of individual fitness level.

#### 2.2.4 Test

The participants’ memory for the series of pictures was assessed. The participants were presented with one cue picture and two other pictures, among which they had to select the one belonging to the same series as the cue picture. The two options could represent the immediate next item in the series (direct trials) or could be separated by one or two items from the cue picture [inference of order 1 or order 2 trials; Fig. 1(c)]. All types of trials were shown in a randomized order and were presented in the same format as during learning, with the exception that no feedback was shown.

### 2.3 NIRS Measurements

#### 2.3.1 Data acquisition

For this study, we used the Oxymon MKIII device (Artinis Medical Systems B.V., Elst, the Netherlands) with eight optodes combined in an overlapping fashion to form six channels [see full lines on Fig. 1(d)]. Five out of six channels were placed at an interoptode distance of 35 mm, and one channel was set at 15 mm (short separation channel) to subsequently remove effects of sudation and capillary blood flow increase in the superficial layers of the head^32^ (scalp and skull, see Sec. 2.3.2). For all the NIRS measurements, the used interoptode distances and optode placement was consistent with the literature for recording of brain hemodynamics during aerobic exercise.^33^^,^^34^

The optode holder was placed on the forehead according to the EEG 10-20 system, so that Fpz was at the exact center of the square formed by the holder [Fig. 1(d)]. Having a fixed optode-holder, the rest of the optodes fell into place as described above. These positions allowed us to record blood oxygenation level-dependent activity over the medial prefrontal cortex but avoiding main facial vessels [avoiding the supraorbital vein and discarding the channel that goes over the frontal branch of the superficial temporal vessels, see dashed line Fig. 1(d)]. The sampling rate was 10 Hz, corresponding to the literature standards^35^^,^^36^ and measurements were biased to zero at the beginning of both the exercise and the rest periods to track only hemoglobin level changes.

#### 2.3.2 NIRS analysis

Data were preprocessed using standard procedures as implemented in Homer2 (MGH-Martinos Center for Biomedical Imaging, Massachusetts).^37^ Motion artifacts were defined as signal change of 0.1 or higher in optical density units over 0.5 s. These artifacts, plus the 0.5 s before and after them, were removed from the analysis. We then applied a low-pass filter of 0.5 Hz to remove the heart beat and respiration effects. We performed a short channel separation correction to remove any effects from scalp and skull, such as sudation and superficial vasodilation from the five deeper channels. This correction was done using a third-order polynomial regressor to extract the signal coming from the superficial layers of the head (picked up by the short separation channel) from the rest of the channels.^38^ All analyses were performed on the ${\mathrm{HbO}}_{2}$ and HHb signals. Because it is widely accepted that spontaneous oscillations are more prominent in ${\mathrm{HbO}}_{2}$ than in HHb data,^24^^,^^39^^–^^41^ we focused our analyses on ${\mathrm{HbO}}_{2}$ oscillations (see Appendix for HHb results).

Data from the first and last 2.5 min of the exercise and rest periods were excluded to minimize unspecific modulations of signal at the start and end of the pedaling. Mean signal and standard deviation (SD) were extracted. Power spectral density (PSD) was calculated using Welch’s method for the interval between 0.003 and 0.15 Hz, i.e., in the frequency range of vasomotion.^17^ Vasomotion was further subdivided into three sub-bands or components corresponding to different oscillatory sources: endothelial (0.003 to 0.02 Hz), neurogenic (0.02 to 0.04 Hz), and myogenic (0.04 to 0.15 Hz).^41^ In order to make valid comparisons of SDs and PSDs of different vasomotion components between the exercise and rest conditions, the SDs and PSDs of ${\mathrm{HbO}}_{2}$ signal were normalized to their mean values across exercise and rest periods:^24^^,^^39^^,^^40^

$$\mathrm{norm}{\mathrm{SD}}_{\text{exercise}}=2\times {\mathrm{SD}}_{\text{exercise}}/({\mathrm{SD}}_{\text{exercise}}+{\mathrm{SD}}_{\text{rest}}),\;\mathrm{norm}{\mathrm{SD}}_{\text{rest}}=2\times {\mathrm{SD}}_{\text{rest}}/({\mathrm{SD}}_{\text{exercise}}+{\mathrm{SD}}_{\text{rest}}).$$

For the PSDs, the normalization was similar, but calculated for endothelial, neurogenic, and myogenic components separately.

$$\mathrm{norm}{\mathrm{PSD}}_{\text{exercise}}=2\times {\mathrm{PSD}}_{\text{exercise}}/({\mathrm{PSD}}_{\text{exercise}}+{\mathrm{PSD}}_{\text{rest}}),\;\mathrm{norm}{\mathrm{PSD}}_{\text{rest}}=2\times {\mathrm{PSD}}_{\text{rest}}/({\mathrm{PSD}}_{\text{exercise}}+{\mathrm{PSD}}_{\text{rest}}).$$

To be able to compare the different components to each other, normalization was performed relative to the mean of the three components as follows:

$$\mathrm{norm}{\mathrm{PSD}}_{\text{endothelial}}=3\times {\mathrm{PSD}}_{\text{endothelial}}/({\mathrm{PSD}}_{\text{endothelial}}+{\mathrm{PSD}}_{\text{neurogenic}}+{\mathrm{PSD}}_{\text{myogenic}}),\;\mathrm{norm}{\mathrm{PSD}}_{\text{neurogenic}}=3\times {\mathrm{PSD}}_{\text{neurogenic}}/({\mathrm{PSD}}_{\text{endothelial}}+{\mathrm{PSD}}_{\text{neurogenic}}+{\mathrm{PSD}}_{\text{myogenic}}),\;\mathrm{norm}{\mathrm{PSD}}_{\text{myogenic}}=3\times {\mathrm{PSD}}_{\text{myogenic}}/({\mathrm{PSD}}_{\text{endothelial}}+{\mathrm{PSD}}_{\text{neurogenic}}+{\mathrm{PSD}}_{\text{myogenic}}).$$

For all the analyses of variance (ANOVA), we included a channel factor as repeated measure to assess if there was an effect of the different channels on normalized SD and PSD data. We found no main effect of channel and no interaction effect with channel (all p>0.05), so channels were pooled for the subsequent analyses. We performed pairwise t-tests to assess difference between conditions (exercise and rest), and one-way repeated measures ANOVA with components of vasomotion as factor to test for differences between endothelial, neurogenic, and myogenic frequency bands (followed by Bonferroni correction of *post hoc* pairwise comparisons). Correlations with performance were done using Spearman’s correlation on ranks as normal distribution and equal variance criteria were not met in our sample.

### 2.4 Memory Performance Analysis

For the analysis of the associative memory task, a learning criterion was applied so that, during the test phase, we only included the responses to those pictures that had actually been learnt by the participants. We considered that a picture was learnt when during the learning session, the participant correctly chose it at least twice across the three learning blocks. During the test part only the trials where these pictures were the picture to be chosen were included in the analysis. Using this criterion, on average 3.1±2.26 pictures per participant were excluded. All behavioral analyses were performed on accuracy data (percentage of correct responses) and on reaction time data (for correct responses) using Statistica (Version 13, StatSoft, Inc. TULSA, Oklahoma).

## 3 Results

### 3.1 Behavioral Results

#### 3.1.1 Learning

Accuracy and reaction time data were analyzed using two repeated measures ANOVA with learning blocks as factor (block 1, block 2, and block 3). Accuracy analysis revealed a main effect of block [F(2,26)=10.730, p=0.0004] consistent with a progressive learning of the associations. During the third block, the participants reached a high level of performance (79.27±6.78% of correct responses). Importantly, there was no effect of “experimental visit number” [F(1,13)=−0.244, p=0.629] or “visit theme” [F(1,13)=0.334, p=0.572] when those factors were added as repeated measures to the previous ANOVA. There was no difference in reaction times across blocks [F(2,26)=1.924, p=0.166].

#### 3.1.2 Test

**Fig. 2:**
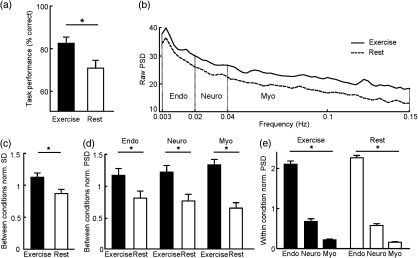
Behavioral and NIRS results. (a) Improved associative memory performance at test after exercise than after rest. (b) Group power spectrum density plot from cerebral ${\mathrm{HbO}}_{2}$. (c) Normalized SD of the ${\mathrm{HbO}}_{2}$ signal is significantly higher during exercise than during rest. (d) Relative mean normalized PSDs between conditions (exercise and rest). (e) Relative mean normalized PSDs within condition.

Accuracy and reaction time data from the test were analyzed using two repeated-measures ANOVAs with condition (exercise and rest) and inferences (direct, inference 1, and inference 2) as repeated measures. For accuracy, we found a significant effect of condition on memory performance [F(1,13)=17.272, p=0.001], but no effect of inference [F(2,26)=3.011, p=0.065; Fig. 2(a)] indicating that the participants perform better after exercise than after rest. For reaction times, there was no effect of condition [F(1,13)=2.640, p=0.128] but an effect of inference [F(2,26)=6.394, p=0.006). *Post-hoc* analysis showed that the participants perform inference 1 and inference 2 trials slower than direct trials ($p_{\text{direct}\text{-}\inf1}=0.012$, $p_{\text{direct}\text{-}\inf2}=0.016$).

#### 3.1.3 Psychomotor vigilance task

We report no difference in PVT data after exercise or after rest (paired t-tests, all p-values >0.05, see Table 1), indicating that the participants were equally vigilant after exercise and after rest.

**Table 1:** Comparison of PVT results after exercise and after rest.

	PVT after exercise	PVT after rest	t-test p-value
Mean reaction times	312.238	311.023	0.907
Median reaction times	295.941	296.118	0.982
Standard error of RT	9.533	10.693	0.652
Number of false alarms	0.118	0.176	0.641
Number of lapses	1.235	1.294	0.911

### 3.2 Heart Rate

Heart rate was 69.3±9.9 at rest and 111.6±17.8 during exercise [t(26)=8.565, p<0.001]. The heart rate observed during exercise corresponded to 58.8±8.5% of participants’ theoretical maximal heart rate. There was no difference in heart rate during the encoding and test sessions, both of which were separated by the exercise or the rest condition [paired t-test $t_{\text{exercise}}(26)=-0.369$, p=0.715; $t_{\text{rest}}(26)=1.457$, p=0.157], and there was no correlation between exercise heart rate and performance on the task (R=−0.0605, p=0.852) or with power in the vasomotion frequency band (R=−0.0649, p=0.841), neither did percentage of AAMHR correlate with these measures (with performance on the task: R=−0.0623, p=0.848; with power in the vasomotion frequency band: R=−1.696, p=0.618).

### 3.3 Body Mass Index and Gender

All participants had a BMI between 18 and 25, except for one who was at 26. There was no significant correlation between BMI and task performance improvement (R=0.267, p=0.378) or power in the vasomotion frequency band ($R_{\text{exercise}}=-0.087$, p=0.778; $R_{\text{rest}}=0.093$, p=0.762).

We ran one-way repeated measures ANOVAs with gender as a factor for all of our measures and found no effect of gender for any of them (all p>0.1).

### 3.4 NIRS

#### 3.4.1 Exercise versus rest

Normalized SD of the ${\mathrm{HbO}}_{2}$ signal was significantly higher during exercise than during rest [t(26)=2.566, p=0.016; Fig. 2(c)] while the mean ${\mathrm{HbO}}_{2}$ signal was not significantly different [t(26)=0.036, p=0.971]. This indicates that the ${\mathrm{HbO}}_{2}$ signal showed significantly higher amplitude fluctuations during the exercise condition. Power spectrum was extracted for every participant to look at the vasomotion-induced oscillations in the frequency range between 0.003 and 0.15 Hz. Figure 2(b) shows the mean power spectrum density for all participants. As shown in Fig. 2(d), the power of all three vasomotion components increased significantly during exercise: endothelial [t(26)=2.086, p=0.047], neurogenic [t(26)=2.957, p=0.007], and myogenic [t(26)=5.331, p<0.001].

#### 3.4.2 Within condition

One-way repeated measures ANOVAs were run for each condition (exercise and rest) separately and with the component of vasomotion as a factor. This yielded a significant effect of component for each condition [exercise: F(2,26)=380.27, p<0.001; rest: F(2,26)=347.45, p<0.001). Bonferroni *post hoc* analysis revealed a predominant effect of the endothelial component in vasomotion, followed by the neurogenic component, and the myogenic component (*post hoc* effects: $\text{endothelial}\text{-}{\text{neurogenic}}_{\text{exercise}}$: p<0.001, $\text{endothelial}\text{-}{\text{myogenic}}_{\text{exercise}}$: p<0.001, $\text{neurogenic}\text{-}{\text{myogenic}}_{\text{exercise}}$: p<0.001; $\text{endothelial}\text{-}{\text{neurogenic}}_{\text{rest}}$: p<0.001, $\text{endothelial}\text{-}{\text{myogenic}}_{\text{rest}}$: p<0.001, $\text{neurogenic}\text{-}{\text{myogenic}}_{\text{rest}}$: p<0.001) [Fig. 2(e)].

**Fig. 3:**
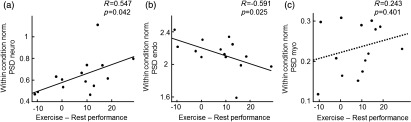
Correlation between memory consolidation and ${\mathrm{HbO}}_{2}$ vasomotion during exercise. (a) Performance enhancement due to exercise correlated positively with PSD in the neurogenic component. (b) Performance enhancement due to exercise correlated negatively with PSD in the endothelial component. (c) Performance enhancement due to exercise did not correlate with PSD in the myogenic component.

To test for a link between vasomotion and memory consolidation, we performed correlations between each component of vasomotion (normalized within condition) during physical exercise and exercise-related performance improvement (${\text{performance}}_{\text{exercise}}\text{-}{\text{performance}}_{\text{rest}}$). We observed a significantly positive relationship between the neurogenic component and performance improvement [R=0.547, p=0.043; Fig. 3(a)], and a significantly negative correlation between the endothelial component and performance improvement [R=−0.591, p=0.026; Fig. 3(b)]. No correlation was found with the myogenic component [R=0.243, p=0.401; Fig. 3(c)].

## 4 Discussion

In this study, we show (1) that 30 min of medium intense exercise enhances the consolidation of associative memories; (2) that the SD of the ${\mathrm{HbO}}_{2}$ signal and the power over all three components of vasomotion (endothelial, neurogenic, and myogenic) are higher during exercise as compared to rest; and (3) that memory performance increase due to exercise correlates positively with the neurogenic vasomotion component and negatively with the endothelial component. Overall these results suggest that modulations of vasomotor activity over the prefrontal cortex during exercise are associated with enhanced memory consolidation.

### 4.1 Better Memory Performance After Exercise

The increase in performance after exercise as compared to after rest is in line with recent results by Van Dongen et al.^11^ showing that high intensity exercise has a positive effect on memory consolidation and memory coding in the hippocampus (tested 48 h after exercise) but only if exercising occurred 4 h after initial learning. Our results demonstrate that memory effects may also be seen for medium intensity exercise, and when the latter takes place immediately after learning. Previous research on exercise has also highlighted the effect of acute exercise on executive functions,^15^^,^^35^^,^^42^ and we know that memory performance may be enhanced by increased attention and vigilance.^43^ In our study, we did not find any difference in PVT measures after exercise from after rest (Table 1), suggesting that the observed beneficial effect of exercise on memory was not primarily driven enhanced attention or vigilance. We also sought to assess whether participant’s individual fitness level influenced their performance. Since exercise level was constant for all participants in our study, we estimated fitness levels from the percentage of AAMHR heart rate measures (detailed in Sec. 2.2.3). We found no correlations with performance, which overall suggests that it is unlikely that individual fitness level is a major contributor to the memory consolidation enhancement we report here.

### 4.2 Increased Vasomotion During Exercise

For the NIRS measurements, a short separation channel was included to record extracranial signal and correct for sudation-related artifacts and cutaneous venous contribution including systemic scalp effects.^44^^–^^47^ Although cerebral systemic effects may differ from scalp systemic effects, it is acknowledged that slow oscillations in the vasomotion frequency bands, that we analyzed here, are less contaminated by systemic effects as for example Mayer waves (0.1 Hz) or hemodynamic response functions.^38^ It has been suggested that the short channel location has an important role, as superficial artifacts are not homogeneously distributed, but are rather localized in the scalp draining veins.^48^ Ideally, we should have included as many short channels as regular channels in our design,^49^ but this was unfortunately not possible with our equipment. However, we carefully positioned all our channels so that they did not cross main arteries or veins. Moreover, we reported no effect of channel for any of our ${\mathrm{HbO}}_{2}$ measures, which further supports that our optode placement and short channel correction was efficient.

When comparing exercise and rest, we found no difference in the overall mean of ${\mathrm{HbO}}_{2}$ signal, consistent with previous findings demonstrating constant global brain blood flow during low-to-moderate exercise in healthy humans,^50^ see meta-analysis in Ref. 51. By contrast, we reported increased power for all three vasomotion components during the exercise condition (compared to rest). This increase in power is most likely linked to vasodilation^52^ and, hence, to increased oxygenation, which is largely supported by previous findings.^16^^,^^51^^,^^53^^,^^54^ We also found that, in both conditions, the endothelial component was predominant, followed by the neurogenic component, and by the myogenic component [Fig. 2(d)]. This general profile is reminiscent of what was previously reported for light sleep, slow wave sleep, and REM in healthy subjects.^24^ It has been suggested that the endothelial frequency range is the most effective for cerebral autoregulation,^55^ which may explain why endothelial component is predominant across different physiological states, including exercise.

### 4.3 Relating Vasomotor Activity to Memory Consolidation

This is the first time to our knowledge that vasomotor activity during physical exercise has been linked to improvement in memory. Here, we present evidence that 30 min of medium intensity exercise enhances associative memory consolidation and that this effect likely relates to changes in vasomotion especially in the neurogenic and endothelial components over the prefrontal cortex. Although relatively little is known about the effects of vasomotion on cognition, a recent finding suggests that vasomotion is impaired in Alzheimer’s disease.^23^ On one hand, moderate physical exercise is known to increase glucose uptake in the brain,^56^ on the other hand, power in the neurogenic component and glucose uptake have been directly linked.^57^ Based on these observations, we would like to propose that, in our study, higher neurogenic activity during exercise may be associated with increased glucose uptake levels over prefrontal cortices, which in turn would boost memory consolidation processes.^58^

More generally, we show that near-infrared spectroscopy may be a valuable method for investigating brain plasticity during physical exercise. Finally, our results may challenge and refine models of memory consolidation that generally focus on rest or sleep periods while we show that an active period of physical exercise may yield similar improvements in memory.

## Figures and Tables

**Fig. 4:**
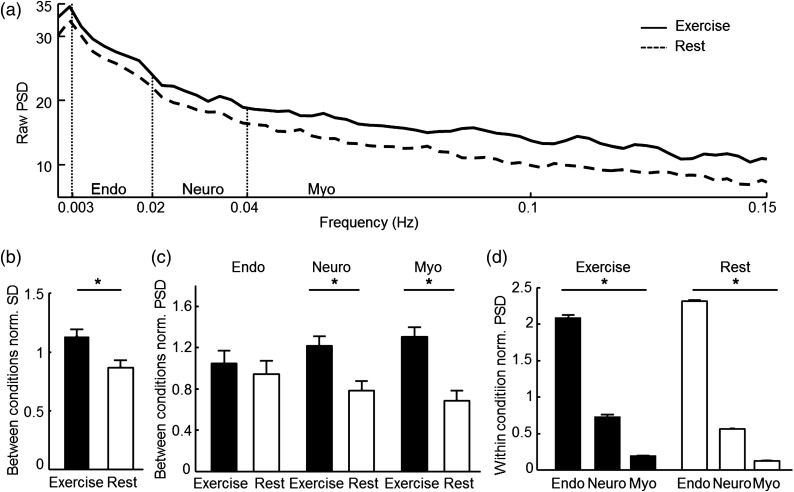
HHb NIRS results. (a) Group power spectrum density plot from cerebral HHb (b) Normalized SD of the HHb signal was significantly higher during exercise than during rest. (c) Relative mean normalized PSDs between conditions (exercise and rest). (d) Relative mean normalized PSDs within condition.

## Appendix A: HHb Results

### A.1 Analysis of HHb Signal between Conditions

Normalized SD of the HHb signal was significantly higher during exercise than during rest [t(26)=2.965, p=0.006, see Fig. 4(b)] while the mean HHb signal was not significantly different between conditions [t(26)=−0.537, p=0.596]. This indicates significantly higher amplitude in the HHb signal during the exercise condition.

Power spectrum was extracted for every subject to assess the vasomotion-induced oscillations in the frequency range of 0.003 to 0.15 Hz. Figure 4(a) shows the mean power spectrum density for all subjects. As shown in Fig. 4(c), the power of the neurogenic and myogenic components increased significantly during exercise {neurogenic [t(26)=3.173, p=0.004], myogenic [t(26)=4.682, p<0.001]}, although power in the endothelial component did not differ {endothelial [t(26)=0.564, p=0.577]}.

### A.2 Analysis of HHb Signal within Conditions

After normalization across exercise and rest condition, we report an effect of component for both conditions [exercise condition: F(2,26)=38.498, p<0.001; rest condition: F(2,26)=721.83, p<0.001, Fig. 4(d)]. Bonferroni *post hoc* analysis shows a predominant effect of the endothelial component in vasomotion (*post hoc* effects: endothelial-neurogenic: p<0.001, endothelial-myogenic: p<0.001). The neurogenic component was significantly higher than the myogenic component for the rest condition but only trended toward significance in the exercise condition (neurogenic-myogenic: $p_{\text{rest}}<0.001$, $p_{\text{exercise}}=0.073$).

No significant correlations were found between task performance and any of the components of vasomotion (endothelial: R=−0.225, p=0.459; neurogenic: R=0.214, p=0.482; myogenic: R=0.165, p=0.590).
